# miR-22 targets YWHAZ to inhibit metastasis of hepatocellular carcinoma and its down-regulation predicts a poor survival

**DOI:** 10.18632/oncotarget.13037

**Published:** 2016-11-03

**Authors:** Ming Chen, Wei Hu, Chen-Ling Xiong, Zhen Qu, Chang-Qing Yin, Yu-Hui Wang, Chang-Liang Luo, Qing Guan, Chun-Hui Yuan, Fu-Bing Wang

**Affiliations:** ^1^ Department of Blood Transfusion, Zhongnan Hospital of Wuhan University, Wuchang District, Wuhan 430071, P.R. China; ^2^ Department of Laboratory Medicine, Zhongnan Hospital of Wuhan University, Wuchang District, Wuhan 430071, P.R. China; ^3^ Department of Immunology, School of Basic Medical Sciences, Wuhan University, Wuchang District, Wuhan 430071, P.R. China; ^4^ Guangdong Food and Drug Vocational College, Guangzhou 510520, P.R. China

**Keywords:** miR-22, hepatocellular carcinoma, survival, YWHAZ, FOXO3a

## Abstract

Many miRNAs are associated with the carcinogenesis of hepatocellular carcinoma (HCC) and some exhibit potential prognostic value. In this study, to further confirm the prognostic value of miRNAs in HCC, we employed miRNA-sequencing data of tumor tissues of 372 HCC patients released by The Cancer Genome Atlas (TCGA) and identified 3 miRNAs including miR-22, miR-9-1 and miR-9-2 could be used as independent predictors for HCC prognostic evaluation. As a tumor-suppressive miRNA, miR-22 was down-regulated in HCC tissues. This down-regulation correlated with tumor vascular invasion, Edmondson–Steiner grade, TNM stage, and AFP level. Moreover, biofunctional investigations revealed that miR-22 significantly attenuated cellular proliferation, migration and invasion of HCC cells. Additionally, through gene expression profiles and bioinformatics analysis, YWHAZ was identified to be a direct target of miR-22 and its overexpression partially counteracted the inhibitory effects of miR-22 on HCC cells. Finally, molecular studies further confirmed that miR-22 promoted the accumulation of FOXO3a in nucleus and subsequently reversed invasive phenotype of HCC cells by repressing YWHAZ-mediated AKT phosphorylation. Taken together, these data demonstrate that miR-22 exhibits tumor-suppressive effects in HCC cells by regulating YWHAZ/AKT/FOXO3a signaling and might be used as an independent prognostic indicator for HCC patients.

## INTRODUCTION

A steadily growing number of studies have confirmed that miRNAs play pivotal roles in tumorigenesis, either acting as oncogenes or tumor suppressors to modulate growth, angiogenesis, drug or chemo-resistance, invasion and metastasis of malignant cells [1, 2]. Deregulation of miRNA is essential to keep the malignant phenotype of cancer cells [3–5] and exhibits a specific miRNA expression signature (miRNome) in different cancers, which characterizes the malignant state and defines some of their clinicopathological features [1, 6]. Thus, the distorted and unique expression profile of miRNAs potentiates their usage as sensitive biomarkers for clinical diagnosis and prognosis of cancers.

Hepatocellular carcinoma (HCC) is the fifth most common cancer worldwide and over 50% occurs in China [7, 8]. However, due to a high incidence of recurrence, the prognosis for HCC is very poor (overall ratio of mortality to incidence is 0.95) and it ranks the second causes of cancer-related deaths worldwide in 2012 [8]. For now, it has been suggested that miRNAs play a critical role in regulating tumorigenesis and metastasis of HCC [9–11], and some of them have been characterized to correlate with prognosis or accepted as potential therapeutic targets [12, 13]. For instance, down-regulation of miR-26a in HCC patients is an independent predictor of poor survival [14–17]. Enforced expression of miR-26a suppressed tumor angiogenesis, growth and metastasis of HCC cells through multiple pathways, including HGF-cMet signaling [14], IL-6-Stat3 signaling [15] or directly targeting cyclins [16]. Furthermore, systemic administration of miR-26a in a mouse HCC model dramatically suppressed tumorigenesis and protected mice from disease progression without toxicity [16].

However, owing to the complexity of miRNA-mRNA interactions [18], and the emerging of competing endogenous RNAs (ceRNAs) [19], the role and clinical value of miRNAs in HCC are still far from being clarified. Acting as a tumor-suppressor, miR-22 suppressed tumor growth and metastasis in different cancers, including breast cancer, lung cancer and colorectal cancer [20–23], and also has therapeutic potential in acute myeloid leukemia [24]. Though miR-22 was shown to be down-regulated in HCC patients, its exact role is still controversial. Jiang et al [25] demonstrated that miR-22 promoted HBV-related HCC development through down-regulation of ERα expression, thus attenuating the protective effect of estrogen and causing higher IL-1α expression. However, in other studies, miR-22 inhibited proliferation of HCC cells both *in vivo* and *in vitro* through targeting multiple proteins, including HDAC4, CDKN1A and CCNA2 [26–28]. Hence, a systematic miRNA-seq evaluation for the prognostic value and the function of miRNAs in HCC development is imperative, given that such study may not only implicate miRNAs as prognostic markers, but also reveal potential therapeutic targets.

In this study, by analyzing the miRNA-sequencing data of 372 HCC tissue samples and 49 normal adjacent tissues, the prognostic values of 48 miRNAs were evaluated based on the patient's clinicopathological information provided by The Cancer Genome Atlas (TCGA). Among these 48 miRNAs, miR-22, miR-9-1 and miR-9-2 were significantly decreased or increased in HCC samples and independently predicted overall poor survival of HCC patients. As a tumor suppressor, miR-22 was proved to attenuate cell proliferation, migration and invasion of HCC cells via directly inhibiting YWHAZ expression. Molecular mechanisms analysis further revealed that miR-22 promoted the accumulation of FOXO3a in nucleus by inhibiting YWHAZ-mediated AKT phosphorylation, and subsequently reversed invasive phenotype of HCC cells. These data suggested a novel mechanism by which miR-22 exhibits tumor-suppressive effects in HCC cells and miR-22 might be used as an independent prognostic indicator for HCC patients.

## RESULTS

### miR-22 is an independent predictor of overall survival of HCC patients

**Figure 1:**
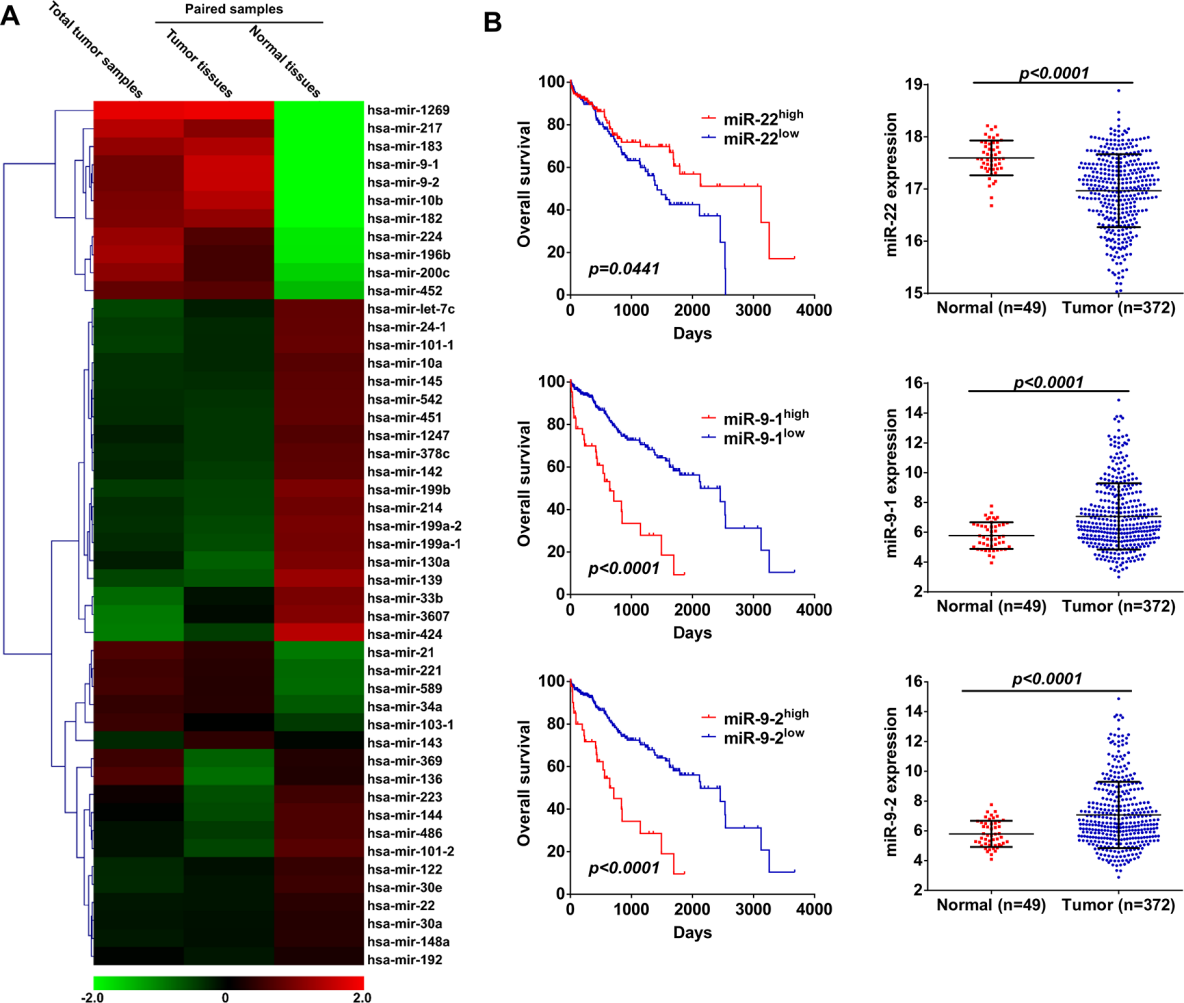
miR-22, miR-9-1 and miR-9-2 are correlated with overall survival of HCC patients (**A**) Expression heatmap of miRNAs in HCC patients with an average of ≥ 10 TPM that either increased or decreased two times between adjacent normal tissues and tumor tissues, and also the the first 10 miRNAs according to the expression abundance either in adjacent normal tissues or tumor tissues was revealed by miRNA-seq data provided by TCGA. (**B**) Kaplan-Meier curves for overall survival according to miR-22, miR-9-1 and miR-9-2 expression levels in HCC patients, cutoff value is the average expression level (Left panel). *p*-value was calculated based on log rank test. Expression of miR-22, miR-9-1 and miR-9-2 in tumor tissues and adjacent normal tissues was revealed by miRNA-seq data provided by TCGA (Right panel).

To identify miRNAs with prognosis potential in HCC, we firstly analyzed next-generation miRNA-sequencing (miR-seq) data of HCC patients that provided by TCGA data portal. A total of 1046 known miRNAs were detected in tumor tissues of 372 HCC patients and adjacent normal tissues of 49 patients. Among the 202 miRNAs with an average of ≥ 10 TPM (transcript per million) after exclusion of the 844 poorly expressed miRNAs with an average TPM of < 10 [29], we identified 41 significantly differentially expressed miRNAs that either were increased or decreased by two times between the two groups (Figure 1A and Supplementary Figure S1). Among these identified miRNAs, the expression of 6 miRNAs was confirmed to be correlated with overall survival of HCC patients, including miR-223, miR-139, miR-33b, miR-21, miR-9-1, miR-9-2 (Figure 1B and Supplementary Figure S2). In addition, individual miRNA abundance is also an important parameter for miRNA-based evaluation of disease progression [30], we then further evaluated the prognostic value of the first 10 miRNAs according to the expression abundance either in tumor tissues or normal tissues (Supplementary Table S1). Among these miRNAs, miR-22 was down-regulated in HCC tissues and predicted poor overall survival of HCC patients (Figure 1B).

Together with miR-22, 7 miRNAs were confirmed to be correlated with overall survival of HCC patients. In order to further confirm whether these 7 identified miRNAs could be used as independent predictor for prognosis evaluation of HCC patients, univariate and multivariate analyses were performed in the cox proportional hazard regression model with simultaneously included clinicopathological characteristics (Table 1). Univariate analysis demonstrated that age, virus infection and 7 miRNAs were significantly associated with overall survival. When the data were stratified for multivariate analysis using both forward and backward stepwise cox regression procedures, only miR-22, miR-9-1 and miR-9-2 remained statistically significant (Table 1), suggesting that these 3 miRNAs are independent predictors for overall survival of HCC patients.

Correlation analysis between expression of these 3 miRNAs and clinicopathological characteristics showed that miR-22 expression was significantly associated with age, Edmondson-Steiner grade, TNM stage, AFP (alphafetoprotein) level and vascular invasion (Supplementary Table S2). However, miR-9-1 and miR-9-2 only associated with vascular invasion (Supplementary Table S2), we thus focused our efforts to evaluate the role of miR-22 in HCC.

**Table 1:** Multivariate analysis using the forward stepwise Cox regression procedure

Variables	Univariate^a^	Multivariate^b^		
	*p*-value	HR	95% CI	*p* value
Age (years)				
≥ 60 *vs* < 60	0.0306*			0.2006
Gender				
Female *vs* Male	0.8376			0.5985
Edmondson–Steiner grade				
G1-2 *vs* G3-4	0.3593			0.2302
TNM stage				
I–II *vs* III–IV	0.1141	1.9232	1.0347–3.5748	0.0387*
AFP level (ng/ml)				
≥ 400 *vs* < 400	0.7592			0.6320
Alcohol consumption				
Yes *vs* No	0.3093			0.2393
HBV/HCV infection				
Yes *vs* No	0.0060*			0.6725
Vascular invasion				
None *vs* Micro/macro	0.3129			0.1351
miR-33b expression				
High *vs* low	0.0070*			0.0871
miR-9-1 expression				
High *vs* low	< 0.0001*	4.1143	2.1100–8.0225	< 0.0001*
miR-9-2 expression				
High *vs* low	< 0.0001*	3.6734	1.856–7.2687	< 0.0001*
miR-21 expression				
High *vs* low	0.0054*			0.2933
miR-22 expression				
High *vs* low	0.0411*	0.4584	0.2514–0.8359	0.0109*
miR-223 expression				
High *vs* low	0.0421*			0.3171
miR-139 expression				
High *vs* low	0.0099*			0.7177

Abbreviations: CI = confidence interval; HR = harzard ratio.

* *p* value less than 0.05 with significant difference between two groups.

a the Kaplan-Meier method, and significance was determined by log-rank test.

b multivariate survival analysis was performed using Cox proportional hazard model.

### miR-22 suppresses migration and invasion of HCC cell lines

**Figure 2:**
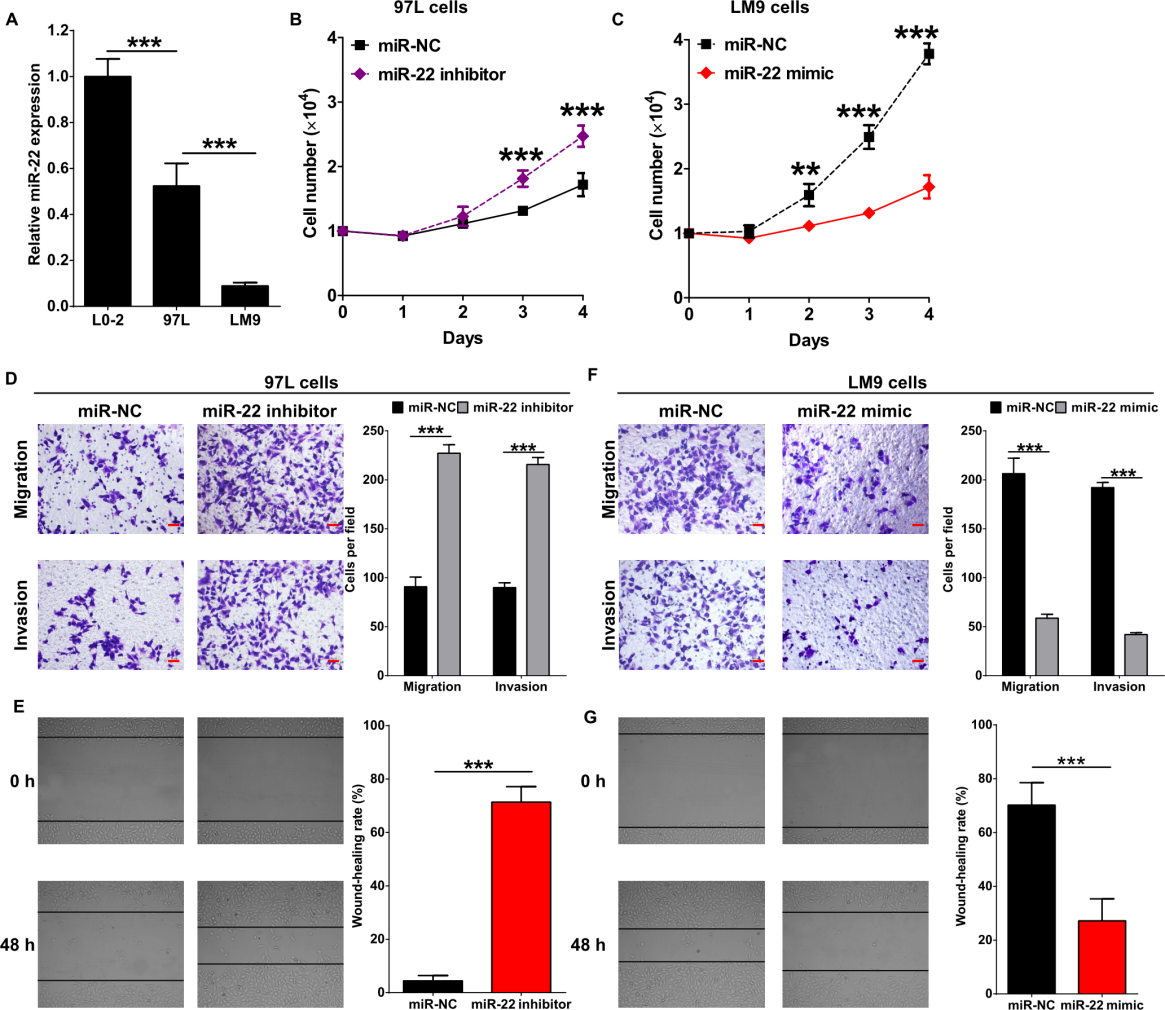
miR-22 inhibits migration and invasion of HCC cells (**A**) miR-22 expression in normal liver cell line L0-2 and HCC cell lines MHCC97L and HCCLM9 was determined by qRT-PCR. Numbers of MHCC97L cells (**B**) and HCCLM9 cells (**C**) were counted at indicated time post transfected with miR-22 inhibitor, miR-22 mimic or miR-22 negative control (miR-NC). Cellular migration and invasion of MHCC97L cells were evaluated by transwell assays (**D**) and wound healing assay (**E**). Cellular migration and invasion of HCCLM9 cells were evaluated by transwell assays (**F**) and wound healing assay (**G**).Quantification of the numbers of migrating or invading cells is presented as mean ± SD from three independent experiments. **p < 0.01, ***p < 0.0001. Scale bar represents 50 μm.

It was reported that miR-22 inhibits tumor metastasis by directly targeting ATP citrate lyase in osteosarcoma, prostate cancer, cervical cancer and lung cancer [23]. Here, we analyzed the expression of miR-22 in the human HCC cell lines with different metastatic potential, MHCC97L and HCCLM9. The level of miR-22 was significantly decreased in both MHCC97L and HCCLM9 compared to the non-neoplastic cell line, L0-2 (Figure 2A). Furthermore, level of miR-22 in HCCLM9 cells was lower than which in MHCC97L cells (Figure 2A). Then, MHCC97L cells and HCCLM9 cells were transfected with miR-22 mimic or inhibitor (Supplementary Figure S3), and cellular proliferation was detected before evaluating the role of miR-22 in metastasis of HCC cells. miR-22 inhibitor significantly promoted proliferation of HCC cells and introduction of miR-22 mimic led to an obvious defect in cell proliferation compared to the miR-NC group (Figure 2B and 2C), similar with previous reports [26, 27, 31]. *in vitro* transwell and wound healing assays showed that miR-22 inhibitor vigorously enhanced migration and invasion of MHCC97L cells (Figure 2D and 2E). Accordingly, the treatment of miR-22 mimic resulted in evident suppression of migration and invasion in HCCLM9 cells (Figure 2F and 2G). Thus, the above findings indicate that miR-22 functions as a metastasis suppressor in HCC.

### YWHAZ is a direct downstream target of miR-22

**Figure 3:**
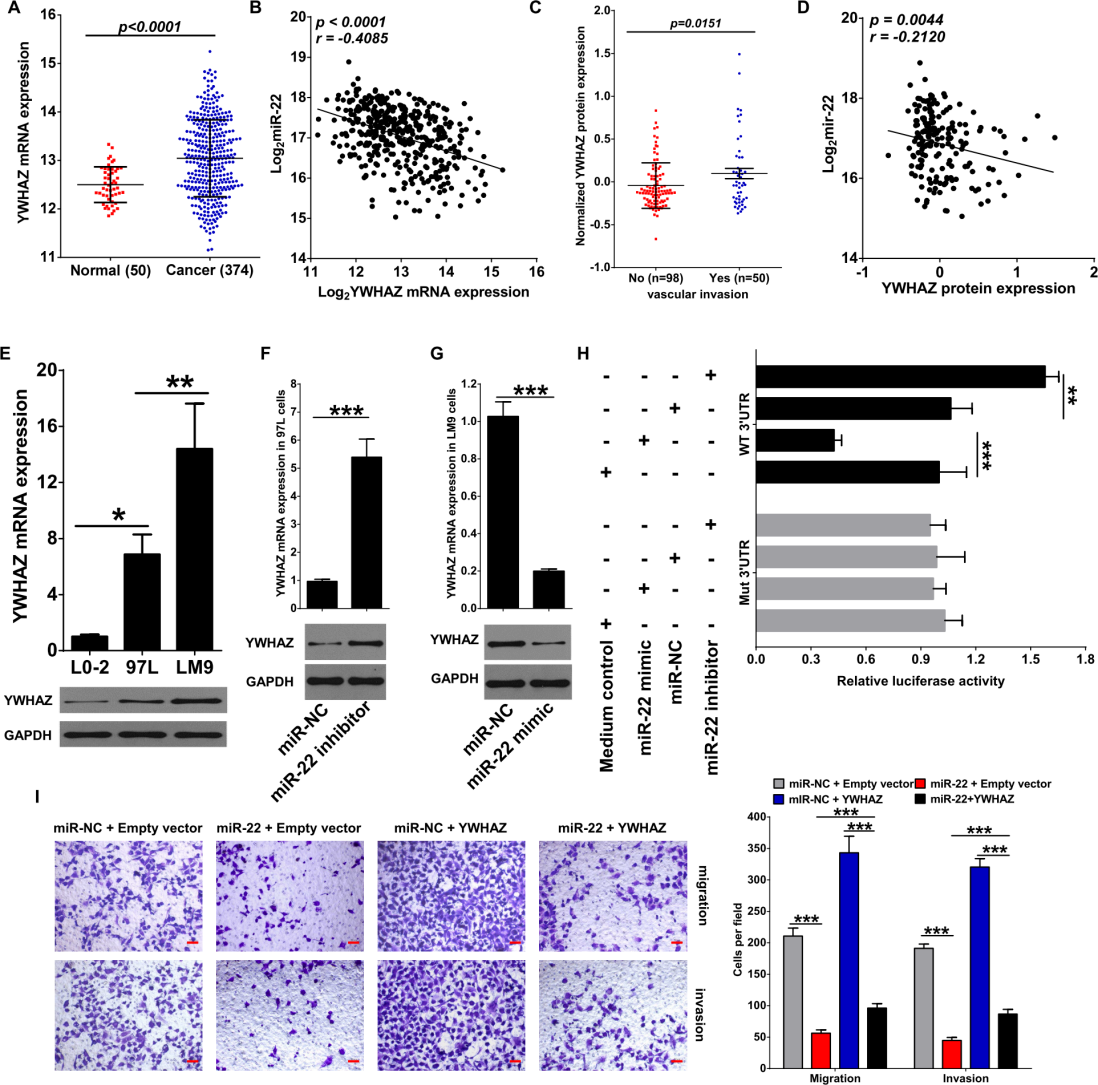
miR-22 inhibits migration and invasion of HCC cells through directly targeting YWHAZ expression (**A**) mRNA expression of YWHAZ in tumor tissues and adjacent normal tissues of HCC patients was revealed by mRNA-seq provided by TCGA. (**B**) Scatterplot depicts a significant inverse correlation between miR-22 and YWHAZ mRNA expression. (**C**) Normalized protein expression of YWHAZ in HCC patients with or without vascular invasion revealed by Reverse Phase Protein Array (RPPA) analysis. (**D**) Scatterplot depicts a significant inverse correlation between miR-22 and YWHAZ protein expression. (**E**) YWHAZ expression in normal liver cell line L0-2 and HCC cell lines MHCC97L and HCCLM9 was determined by qRT-PCR and western blotting. GAPDH was used as an internal control. (**F**) Effect of miR-22 inhibitor on YWHAZ expression was determined in MHCC97L cells. (**G**) Effect of miR-22 mimic on YWHAZ expression was determined in HCCLM9 cells. (**H**) miR-22 mimic significantly suppressed luciferase activity of YWHAZ containing a wild-type 3′-UTR, but showed no effect on activity of YWHAZ with a mutant 3′-UTR, whereas treatment with miR-22 inhibitor increased luciferase activity of YWHAZ. (**I**) Overexpression of YWHAZ partially rescued the inhibitory effects of miR-22 on migration and invasion of HCC cells. Scale bar represents 50 μm. Data are displayed as the Mean ± SD of three independent experiments. *p < 0.05; **p < 0.01; ***p < 0.0001. (miR-NC: miR-22 negative control, WT: wild type; Mut: mutant type).

To identify which miR-22 targets were responsible for its effects on cancer cell migration and invasion, we predicted the function of miR-22 by starBASE v2.0 with very high stringency enrichment analysis (http://starbase.sysu.edu.cn) [32] and found 63 genes predicted by targetScan, picTar, RNA22, PITA and miRanda were potential targets of miR-22 (data not shown). Among the 63 potential targets, AKT3, CDKN1A, DDIT4, GNB4, NRAS and YWHAZ are the signal proteins of PI3K/AKT pathway and involves in HCV/HBV infection, as well as regulates multiple cancer progression in section of KEGG pathway analysis (Supplementary Table S3). We then analyzed the mRNA expression of these 6 genes in RNA-seq data of HCC patients that provided by TCGA data portal. Interestingly, we found that only NRAS and YWHAZ were elevated in cancer tissues compared to adjacent normal tissues (Figure S4A and Figure 3A). In addition, correlation analysis further confirmed that NRAS and YWHAZ inversely correlated with miR-22 expression in HCC patients (Supplementary Figure S4B and Figure 3B). The relationship between NRAS/YWHAZ and clinicopathological characteristics was summarized in Table 2. We noticed that NRAS and YWHAZ were significantly associated with Edmondson-Steiner grade, no matter in mRNA or protein level. More importantly, YWHAZ protein expression further associated with vascular invasion and also negatively correlated with miR-22 expression (Figure 3C and 3D), while NRAS was not (data not shown).

**Table 2:** Clinicopathological characteristics of HCC patients according to NRAS and YWHAZ expression

Characteristics	NRAS mRNA expression (Mean ± SD)	*p* value	YWHAZ mRNA expression (Mean ± SD)	*p* value	NRAS protein expression (Mean ± SD)	*p* value	YWHAZ protein expression (Mean ± SD)	*p* value
Age (years)		*0.9688*		0.0343*		0.0051*		*0.7443*
≥ 60	10.41 ± 0.5563		12.97 ± 0.8093		0.03212 ± 0.1406		0.01065 ± 0.3677	
< 60	10.40 ± 0.6507		13.15 ± 0.7579		−0.03000 ± 0.1510		0.02714 ±0.2695	
Gender		*0.6340*		*0.6491*		*0.2213*		*0.0683*
Female	10.38 ± 0.5816		13.03 ± 0.8047		−0.02415 ± 0.1698		0.07746 ± 0.3911	
Male	10.42 ± 0.6102		13.07 ± 0.7845		0.009835 ± 0.1460		−0.01600 ± 0.2923	
Edmondson–Steiner grade		0.0130*		< 0.0001*		0.0002*		0.0111*
G1-2	10.34 ± 0.5769		12.91 ± 0.7726		0.03662 ± 0.1268		−0.02295 ± 0.3068	
G3-4	10.51 ± 0.6300		13.32 ± 0.7524		−0.04835 ± 0.1692		0.1079 ± 0.3628	
TNM stage		*0.1187*		*0.4169*		*0.0702*		*0.2091*
I–II	10.36 ± 0.5898		13.04 ± 0.7955		0.02140 ± 0.1468		−0.0005718 ± 0.3043	
III–IV	10.47 ± 0.6057		13.12 ± 0.8017		-0.02070 ± 0.1446		0.06635 ± 0.3835	
AFP level (ng/ml)		*0.6538*		0.0011*		< 0.0001*		0.0095*
≥ 400	10.40 ± 0.6461		13.29 ± 0.7759		−0.07200 ± 0.1501		0.1445 ± 0.4223	
< 400	10.36 ± 0.5695		12.93 ± 0.7823		0.04388 ± 0.1234		-0.02912 ± 0.2743	
Alcohol consumption		*0.1878*		*0.6499*		*0.4258*		*0.9629*
Yes	10.35 ± 0.5687		13.00 ± 0.8031		0.02566 ± 0.1423		0.01069 ± 0.3494	
No	10.43 ± 0.5989		13.04 ± 0.7736		0.007446 ± 0.1476		0.008242 ± 0.3277	
HBV/HCV infection		*0.9849*		*0.5097*		*0.2440*		*0.4237*
Yes	10.40 ± 0.5671		13.07 ± 0.7635		0.03754 ± 0.1193		−0.02553 ± 0.2648	
No	10.40 ± 0.6132		13.01 ± 0.8081		0.006684 ± 0.1537		0.02321 ± 0.3561	
Vascular invasion		*0.6740*		*0.1856*		*0.0597*		0.0151*
None	10.36 ± 0.6012		12.98 ± 0.7891		0.02779 ± 0.1344		−0.04272 ± 0.2652	
Micro/macro	10.39 ± 0.5886		13.11 ± 0.7999		−0.01938 ± 0.1587		0.09696 ± 0.4233	

Next, we further detected mRNA expression of these 6 proteins in HCC cell lines in response to miR-22 mimic or inhibitor treatment. mRNA expression of NRAS, DDIT4, YWHAZ and CDKN1A were increased in HCC cells (Supplementary Figure S4C and Figure 3E). In addition, contrary to miR-22 expression, both mRNA and protein expressions of YWAHZ in MHCC97L and HCCLM9 cells were relatively increased compared with L0-2 cells (Figure 3E). miR-22 inhibitor vigorously up-regulated all 6 transcripts in MHCC97L and HCCLM9 cells (Supplementary Figure S4D and Figure 3F). With regard to miR-22 mimic treatment, we observed a significant down-regulation of AKT3, CDKN1A, DDIT4, NRAS and YWHAZ mRNA levels in both HCCLM9 and MHCC97L cells (Supplementary Figure S4D and Figure 3G). Though only NRAS and YWHAZ were inversely correlated with miR-22 expression in HCC patients, these results confirm that miR-22 has the potential to inhibit AKT3, CDKN1A, DDIT4, GNB4, NRAS and YWHAZ expression in HCC cells.

We next specifically assessed whether miR-22 could directly regulate YWHAZ mRNA expression by binding to its 3′ UTR. We showed that miR-22 mimic significantly suppressed luciferase activity of YWHAZ only when wild-type (WT) 3′-UTRs were present (Figure 3H). Whereas miR-22 inhibitor treatment increased the luciferase activity of YWHAZ (Figure 3H). Overall, these results demonstrate that mRNA of YWHAZ is a direct target of miR-22 in HCC cells.

### miR-22 reduces cell migration and invasion by targeting YWHAZ

To clarify the importance of YWHAZ for miR-22 -mediated inhibition of cell migration and invasion. HCCLM9 cells were transfected with YWHAZ-expressing plasmid with or without miR-22 mimic. We then analyzed whether YWHAZ overexpression could counteract the effects of miR-22 on cell migration and invasion. Our results showed that the inhibitory effects of miR-22 mimic on cell migration and invasion of HCCLM9 cells could be partially compensated by the additional overexpression of YWHAZ (Figure 3I). These data suggest that YWHAZ down-regulation might be one important cause for the decrease in cell migration and invasion observed upon miR-22 overexpression.

### miR-22 promotes nuclear accumulation of FOXO3a by suppressing YWHAZ-mediated AKT phosphorylation

**Figure 4:**
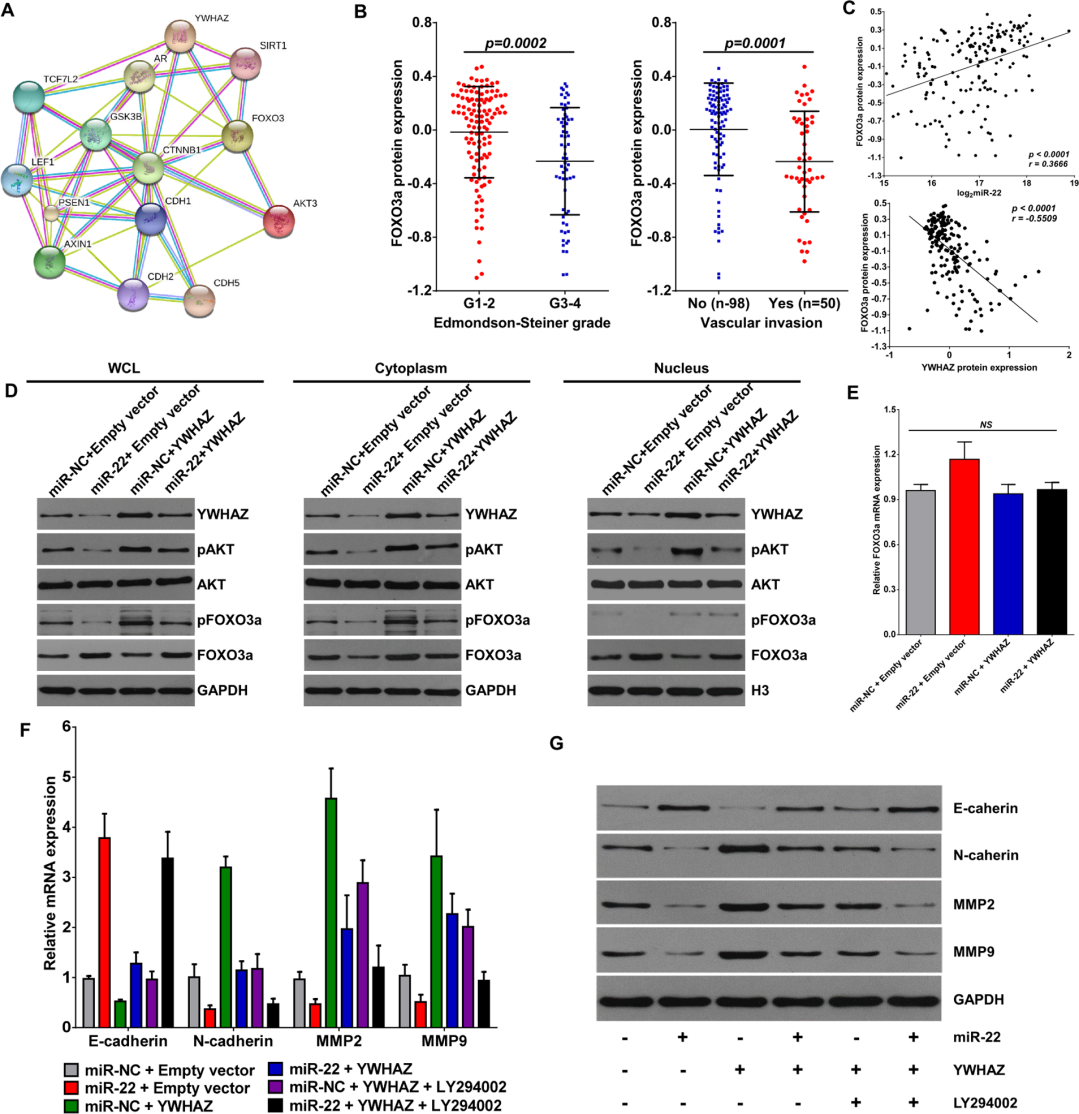
miR-22 promotes the nuclear re-input of FOXO3a via inhibiting YWHAZ expression (**A**) Proteins interacted with AKT3 and YWHAZ was predicted by String database (http://string-db.org). (**B**) FOXO3a protein expression in HCC patients according to Edmondson-Steiner grade and vascular invasion. (**C**) FOXO3a protein expression showed a significant positive correlation with miR-22 and a significant inverse correlation with YWHAZ protein expression in HCC tissues. (**D**) HCCLM9 cells were transfected with miR-22 mimic or pcDNA3.1-YWHAZ recombinant plasmid. 48 h later, protein expression of AKT, pAKT, YWHAZ, pFoxO3a, FOXO3a in different cellular components were detected by western blotting. (**E**) FOXO3a mRNA expression in response to miR-22 mimic and YWHAZ overexpression was determined by qRT-PCR. (**F**) and (**G**) In some cases, HCCLM9 cells were pretreated with LY294002 (PI3K inhibitor, 20 μM) for 1 h, and then transfected with miR-22 mimic or YWHAZ-expressing plasmid for 48 h. mRNA and protein expressions of E-caherin, N-cadherin, MMP2 and MMP9 were detected by qRT-PCR (F) and western blotting (G). Data are displayed as the Mean ± SD of three independent experiments.

As an important tumor-suppressing transcription factor, the activity of FOXO3a can be inhibited by the cooperation between AKT and YWHAZ [33] (Figure 4A). In HCC, the protein expression of FOXO3a was decreased in patients with high Edmondson-Steiner grade and vascular invasion (Figure 4B). Correlation analysis further showed that FOXO3a positively associated with miR-22 expression and negatively with YWHAZ expression (Figure 4C). We next examined the effects of miR-22 and YWHAZ on FOXO3a expression in HCCLM9 cells. Overexpression of YWHAZ caused substantial increase of phosphorylated FOXO3a (p-FOXO3a) accumulation in cytoplasm of HCCLM9 cells, but decreased total FOXO3a expression in nucleus (Figure 4D). However, the effect of YWHAZ on FOXO3a was markedly reversed in the presence of miR-22 mimic (Figure 4D). By detecting AKT and phosphorylated AKT (p-AKT), we further found that miR-22 suppressed YWHAZ-induced p-AKT expression (Figure 4D), which inhibits the activity of FOXO3a by exporting p-FOXO3a from the nucleus and inducing ubiquitination and degradation by the 26S proteasome [33–35]. Furthermore, it was noteworthy that miR-22 alone did not decease total AKT expression and mRNA expression of FOXO3a (Figure 4E) in HCCLM9 cells, but deceased p-AKT, p-FOXO3a, YWHAZ expression and increased total FOXO3a protein expression (Figure 4D). Hence, these results suggest that miR-22 decreases p-FOXO3a expression and promotes nuclear accumulation of FOXO3a mainly through inhibiting YWHAZ-mediated AKT phosphorylation.

FOXO3a suppresses cancer invasiveness through negatively modulating WNT/β-catenin pathway or metastasis related proteins, like snail and twist [36–38]. To further confirm the important role of YWHAZ/FOXO3a signaling in HCC invasion, we detected a panel of key regulators involved in cell migration and metastasis, including E-cadherin, N-cadherin, MMP2 and MMP9. Overexpression of YWHAZ significantly increased both mRNA and protein expression of N-cadherin, MMP2 and MMP9 in HCCLM9 cells, and suppressed E-cadherin expression (Figure 4F, 4G). However, the effect of YWHAZ on these proteins was markedly reversed in the presence of miR-22 or LY-294002, which induces the nuclear accumulation of FOXO3a [39]. And more importantly, miR-22 and LY-294002 showed synergic effects on expression of E-cadherin, N-cadherin, MMP2 and MMP9 (Figure 4F, 4G). Thus, our data demonstrate that FOXO3a is an important indirect downstream molecule of miR-22.

## DISCUSSION

Up to date, dysregulation of multiple miRNAs in HCC tissues have shown potential value in prognostic prediction and cancer therapy, including miR-29 [11], miR-19b [40] and miR-99a [41]. However, with the raise of miRNA signature [1] and the emerging of ceRNAs [19], the certain role and clinical value of miRNAs in HCC remains an ongoing process. In this study, we systematically analyzed miR-seq data of HCC patients and confirmed that three miRNAs are independent prognostic predictors of HCC, including miR-22, miR-9-1, miR-9-2. As a tumor-suppressive and highly abundant hepatic miRNA, miR-22 suppressed migration and invasion of HCC cells via directly targeting YWHAZ.

miR-22 exhibits complicated but important role in the development of numerous cancers [22, 24, 42, 43]. In most cases, restoration of miR-22 expression suppresses cancer progression through targeting multiple oncogenic proteins, like proteins involved in MYC and CREB pathways [22, 24], and exacts therapeutic potential in leukaemic mice model [24]. However, miR-22 is also an epigenetic modifier that promotes stemness and metastasis of myelodysplastic syndrome (MDS) and breast cancer by targeting TET2 [42, 43]. Different studies have confirmed that miR-22 inhibits cellular proliferation [26, 27, 31] and modulates the tumor microenvironment of HCC [44]. In addition, miR-22 also suppresses cancer metastasis by targeting ATP citrate lyase in osteosarcoma, prostate cancer, cervical cancer and lung cancer [23]. However, underlying mechanisms that mediate the suppression of migration and invasion by miR-22 in HCC are still elusive.

Through gene expression profiles and bioinformatics analysis, we confirmed that miR-22 target multiple proteins involved in PI3K/AKT pathway, including AKT3, CDKN1A, DDIT4, GNB4, NRAS and YWHAZ. AKT usually cooperates with YWHAZ to regulate the function of downstream proteins [33, 45]. By detecting AKT phosphorylation in response to miR-22 and YWHAZ treatment, we found that miR-22 significantly decreased phosphorylated AKT level, especially in nucleus, while showed no obvious effect on total protein expression of AKT. In addition, overexpression of YWHAZ enhanced the phosphorylation of AKT, and this effect was counteracted in the presence of miR-22. We speculated that this phenomenon may be resulted by two reasons: 1) miR-22 only specifically target to AKT3 mRNA, and which only occupies a minor part of total mRNA expression of AKT (2.71% ± 3.33%, data not shown); 2) Overexpression of YWHAZ could enhance AKT phosphorylation in cancer cells via binding the p85 regulatory subunit of PI3K [46]. Thus, miR-22 decreases AKT phosphorylation by directly inhibiting YWHAZ expression. Together with the observation that additional overexpression of YWHAZ partially but significantly compensated the inhibitory effects of miR-22 on cell migration and invasion of HCCLM9 cells, so YWHAZ is an important target molecule for miR-22 to suppress metastasis of HCC cells.

Phosphorylated AKT inhibits the activity of FOXO3a mainly by promoting its phosphorylation and binding to YWHAZ, which mediates the nuclear exportation of phosphorylated FOXO3a [47]. In this study, we found that miR-22 decreased p-FOXO3a expression in cytoplasm of HCC cells, and promoted FOXO3a accumulation in nucleus. However, this effect was markedly counteracted in the presence of additional YWHAZ overexpression. Thus, miR-22 decreased the p-FOXO3a by inhibiting YWHAZ-induced AKT phosphorylation and which led to the accumulation of FOXO3a in nucleus of HCC cells. YWHAZ induces hyperactivation of the PI3K/AKT pathway which lead to phosphorylation and translocation of the MDM2 [48]. MDM2 is the E3 ligase that ubiquitinates p-FOXO3a in response to ERK or AKT pathway activation in the cytoplasm [49, 50]. However, YWHAZ overexpression did not decease the expression of FOXO3a and which further increased in the presence of miR-22 in whole cell lysate of HCC cells. This may be resulted by that YWHAZ stabilizes p-FOXO3a in cytoplasm by inhibiting its dephosphorylation and degradation [51]. However, the exact roles of YWHAZ and miR-22 in degradation of FOXO3a by AKT phosphorylation remains need to be clarified in future studies.

**Figure 5:**
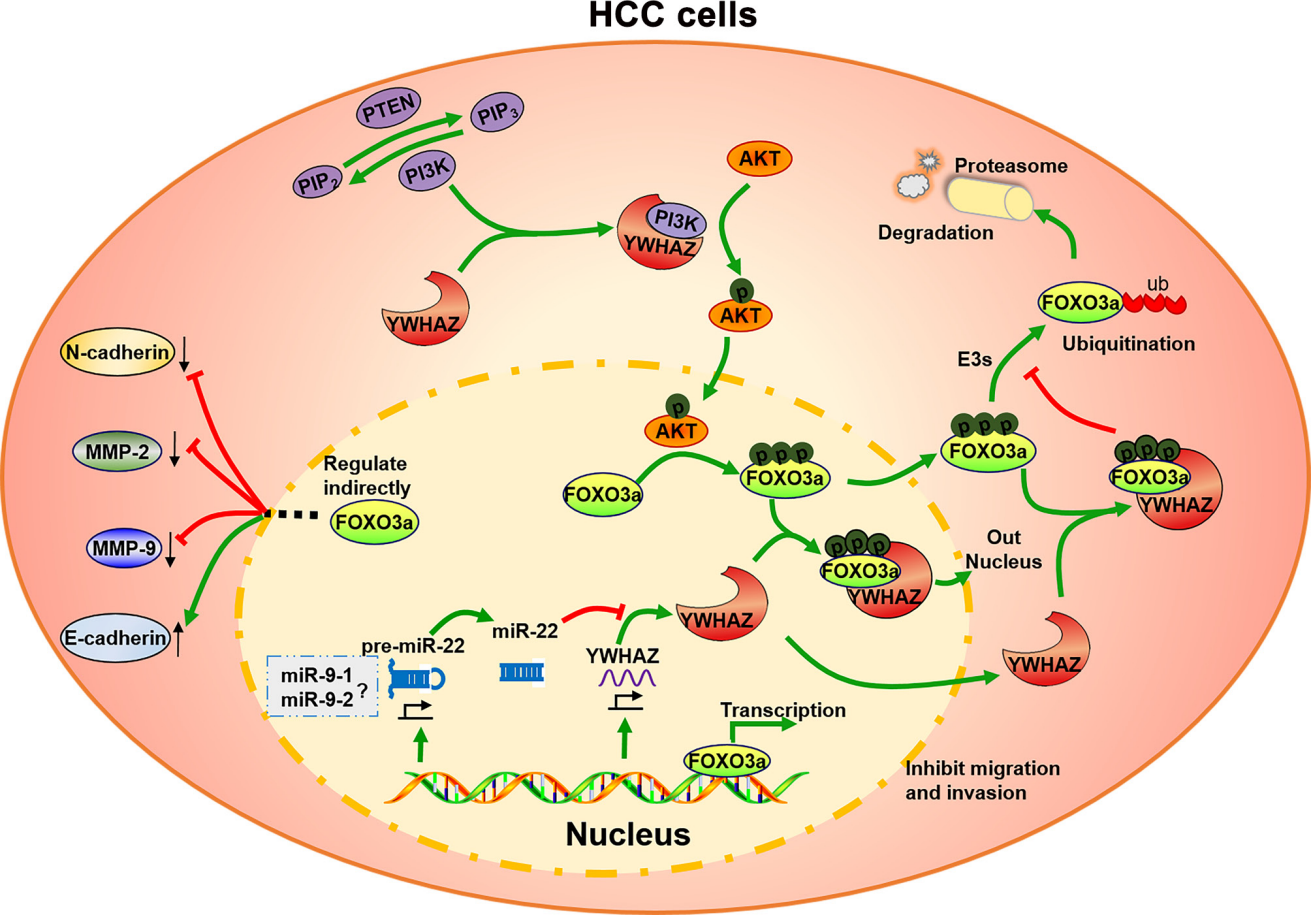
A schematic summary of the findings in this study miR-22 directly targets 3′-UTR of YWHAZ mRNA to inhibit YWHAZ expression. YWHAZ forms a regulatory loop in PI3K/AKT pathway and modulates signaling kinetics. YWHAZ enhances AKT phosphorylation in cancer cells via binding the p85 regulatory subunit of PI3K. And then phosphorylated AKT inhibits the activity of FOXO3a by promoting its phosphorylation and binding to YWHAZ, which mediates the nuclear exportation of phosphorylated FOXO3a. As a tumor-suppressive transcription factor, FOXO3a reverses the invasive phenotype of HCC cells, including down-regulation of N-cadherin, MMP2 and MMP9 expression, up-regulation of E-cadherin expression. Thus, miR-22 exhibits tumor-suppressive effects in HCC cells by regulating YWHAZ/FOXO3a signaling. The question mark means unsolved problems in this study.

In summary, as shown in Figure 5, our results identify three independent prognostic predictor, miR-22, miR-9-1 and miR-9-2 in HCC. Specifically, we further demonstrate that miR-22 inhibits cell migration and invasion via directly inhibiting YWHAZ expression and thus enhances the antitumor activities of the downstream protein, FOXO3a in HCC. The features of this miR-22-YWHAZ-AKT-FOXO3a signaling arm support its exploration may be a therapeutic target or prognostic biomarker for HCC.

## MATERIALS AND METHODS

### TCGA data analysis

Level 3 miRNA expression data (processed/mapped) for 421 HCC specimens profiled using Illumina HiSeq were retrieved from both the miRNA quantification and isoform files available at the TCGA data portal along with metafiles annotating each dataset. Level 3 normalized mRNA expression data for 424 HCC specimens and protein expression data for 368 HCC specimens were profiled using Illumina HiSeq 2000 sequencers and Reverse Phase Protein Array by the Univ. of Texas MD Anderson Cancer Center RPPA Core Lab, respectively. Coded patient survival data was extracted from the TCGA clinical information file. Permission to access all data was obtained from the Data Access Committee for the National Center for Biotechnology Information Genotypes and Phenotypes Database (dbGAP) at the National Institute of Health. The expression of miRNA and mRNA was normalized and presented as Log_2_ value. Analysis of all data was done using GraphPad Prism 6 (San Diego, CA, USA).

### Cell culture and transfection

Normal liver cell lines L0-2 and HCC cell lines MHCC97L and HCCLM9 cells (which have the same genetic background but with step-wise potential metastasize primarily to the lung [52]) were maintained in our laboratory as previously described [52–54] and cultured in RPMI1640 (Gibco) containing 10% FBS (Gibco) and 100 units/mL penicillin-streptomycin (Beyotime, Shanghai, China). Transient transfection was performed using Lipofactamine 2000 (Invitrogen, Shanghai, China) according to the manufacturer's instruction.

### Quantitative real-time PCR (qRT-PCR)

Total RNA was extracted using Trizol (Invitrogen, Shanghai, China) according to the manufacturer's instruction. For miR-22 expression analysis, 10 ng of total RNA from each sample was transcribed into cDNA using TaqMan^®^ MicroRNA Reverse Transcription kit (Applied Biosystems, Shanghai, China). qRT-PCR was performed using TaqMan^®^ 2× Universal PCR Master Mix no UNG (Applied Biosystems, Shanghai, China) and hsa-miR-22 TaqMan primers (Applied Biosystems, Shanghai, China). To analyze mRNA expression, both RNA reverse transcription and qRT-PCR amplification were performed as recommended by ReverTra Ace-α First-strand cDNA Synthesis kit and SYBR Green real-time PCR Master Mix kit (Toyobo, Tokyo, Japan), respectively. The primers used for AKT3, CDKN1A, DDIT4, GNB4, NRAS, YWHAZ and GAPDH are listed in Supplementary Table S4.

### Luciferase assay

DNA sequences containing the putative miR-22 binding site on the 3'UTR of human YWHAZ gene (Genbank accession no. NM_003406) were synthesized (Supplementary Table S4) and cloned into the downstream of the firefly luciferase stop codon in a pmirGLO control vector (Promega, Milan, Italy). HCCLM9 cells were plated in 24 well plates overnight to reach 80% confluence. The following day, the cells were co-transfected with 30 nM of miR-22 mimic, miR-22 inhibitor or miR-NC (Sangon, Shanghai, China) and 500 ng of YWHAZ 3’ UTR (WT or Mut) pmirGLO recombinant vectors. 24 h later, cells were harvested and lysed using a passive lysis buffer (Promega, Milan, Italy). Firefly luciferase activity was detected with Dual Luciferase Assay (Promega, Milan, Italy) according to the manufacturer's instructions and standardized to Renilla luciferase activity as an internal standard. All experiments were performed in triplicate.

### Western blotting

Total cells were lysed in RIPA buffer (Beyotime, Shanghai, China) or treated with NE-PER™ Nuclear and Cytoplasmic Extraction Reagents (Thermo Scientific) according to the product instructions. Next, equal amounts of proteins were separated by SDS-PAGE and then transferred to PVDF membrane (Millipore, MA, USA). After blocking with 5% non-fat dry milk in TBST buffer for 1 h, the membrane was incubated at 4°C overnight with the indicated primary antibodies. Detection was performed by peroxidase-conjugated secondary antibodies and the protein bands were visualized using the enhanced chemiluminescence system (Millipore, MA, USA). The following primary antibodies were used: anti-YWHAZ, anti-MMP2, anti-MMP9 and anti-GAPDH (Abcam, MA, USA), anti-AKT, anti-pAKT, anti-FoxO3a, anti-pFoxO3a, anti-E-cadherin, anti-N-cadherin and anti-Histone H3 (CST, MA, USA).

### Cell proliferation assay

HCC cells were seeded in 96-well plates at the concentration of 1 × 10^4^ cells in 100 μl complete medium per well and then transfected with miR-22 mimic, miR-22 inhibitor or miR-NC. Cell numbers were counted at the indicated days post transfection with cell counting chamber using TC10™ Automated Cell Counter (BioRad, CA, USA) [24].

### Wound healing assay

Cells were plated in a 6-well plate and scratched with a 200-μl pipette tip after achieving nearly 90% confluence. The speed of wound closure was imaged with an inverted microscope (TE-2000, Nikon) at 0 and 48 h.

### *In vitro* cell invasion and migration assays

These experiments were conducted as described previously [52] with some modifications. In brief, Transwell chambers (8 μm pore; Corning Costar) were precoated with 80 μL Matrigel (300 μg/mL; BD Biosciences, USA) and then inserted into the 24-well plate. 24 h after transfection with miRNA or pcDNA3.1-YWHAZ, cells were serum-starved for another 24 h. Next, a total of 5 × 10^4^ cells in 200 μl FBS-free medium were harvested and seeded in the upper chamber. 500 μl medium containing 10% FBS were added in the lower chamber. After 48 h incubation, the invaded cells were fixed with 100% methanol and stained with 0.1% crystal violet (Beyotime), and extensively washed with PBS. Stained cells were then imaged using Nikon TE2000 microscope (× 200 magnification) and counted in four independent areas of the membrane. The migration assay was carried out as described in the invasion assay with no coating of Matrigel and the total cell number is 1 × 10^4^.

### Statistical analysis

All experiments were repeated at least three times. Data are presented as mean ± standard deviation (SD) and analyzed for significance using GraphPad Prism 6 software (San Diego, CA, USA). Difference between two-groups was assessed using student's *t*-test. One-way ANOVA followed by Newman-Keuls post hoc testing (95% confidence) was used to determine difference among more than two groups. The survival analysis was illustrated by Kaplan-Meier curves with log-rank test. Univariate and multivariate survival analyses were performed using the likelihood ratio test of the stratified Cox proportional hazards model of SPSS 17.0 (SPSS Inc. Chicago, IL, USA). *p*-value of < 0.05 is considered statistically significant.

## SUPPLEMENTARY MATERIALS


