# Supplementary material for: miR-22 targets YWHAZ to inhibit metastasis of hepatocellular carcinoma and its down-regulation predicts a poor survival
Source: Oncotarget. 2016 Nov 3;7(49):80751–64. doi: 10.18632/oncotarget.13037 (PMC5348352; doi:10.18632/oncotarget.13037)
Supplement: Supplementary file 1 [file oncotarget-07-80751-s001.pdf]

miR-22 targets YWHAZ to inhibit metastasis of hepatocellular carcinoma and its down-regulation predicts a poor survival

Supplementary Materials

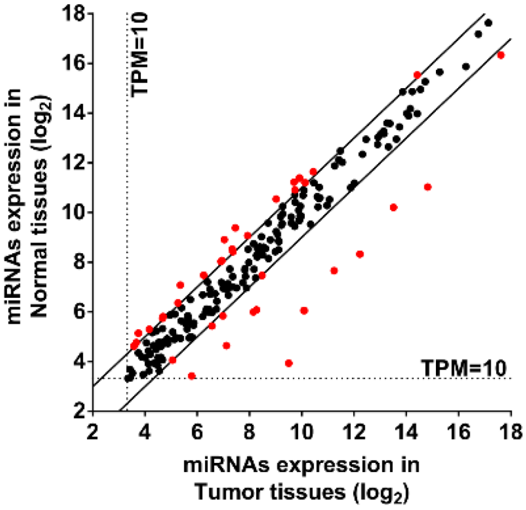

Supplementary Figure S1: Scatterplot depicts the miRNAs expression with an average of ≥ 10 TPM in tumor tissues and adjacent normal tissues of HCC patients. Red points represent the miRNAs either increased or decreased two times between adjacent normal tissues and tumor tissues.

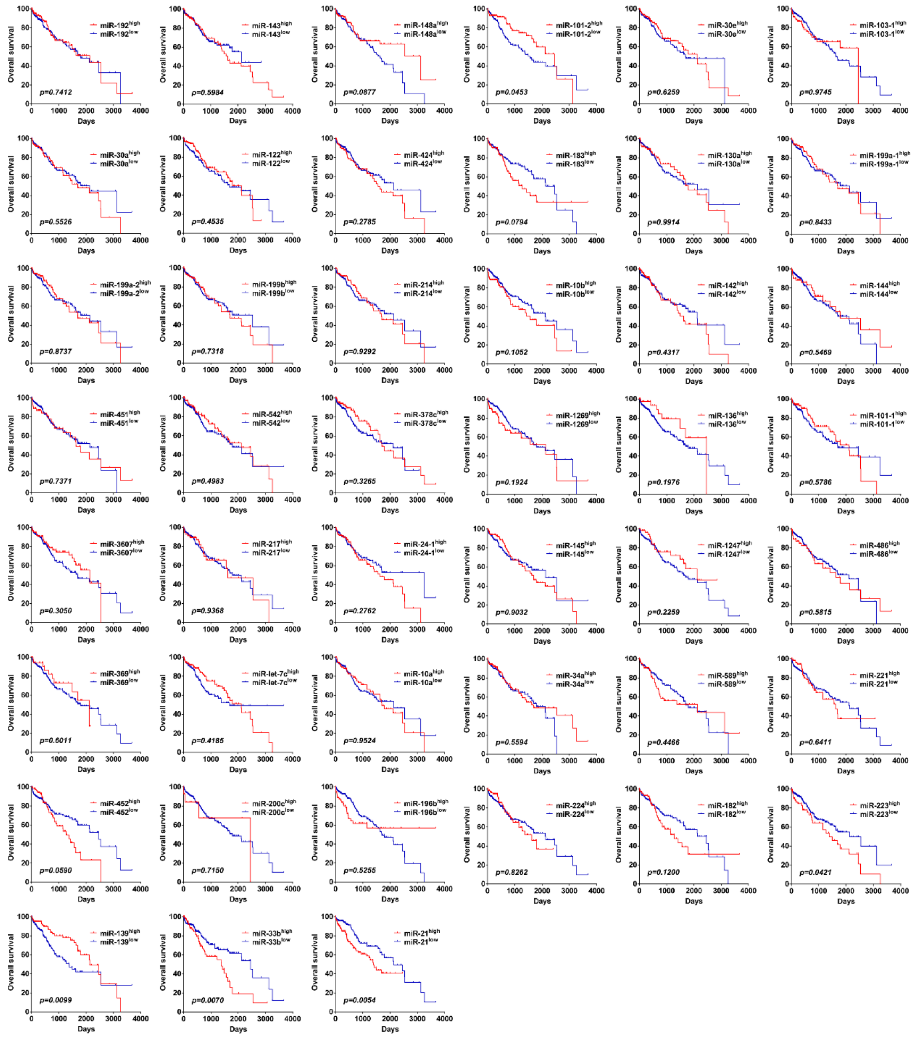

Supplementary Figure S2: Kaplan-Meier curves for overall survival of 45 miRNAs. These 45 miRNAs with an average of ≥ 10 TPM that either increased or decreased two times between adjacent normal tissues and tumor tissues, and also the the first 10 miRNAs according to the expression abundance either in adjacent normal tissues or tumor tissues was revealed by miRNA-seq data provided by TCGA.

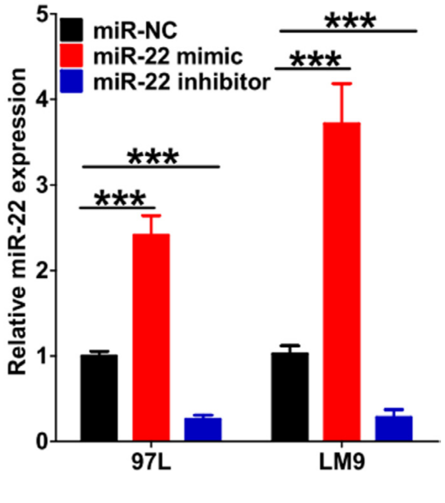

Supplementary Figure S3: The interfering or overexpressing efficiency of miR-22 in HCC cells. MHCC97L cells and HCCLM9 cells were transfected with miR-NC, miR-22 mimic or inhibitor, and miR-22 expression was determined by qRT-PCR 48 h after transfection.

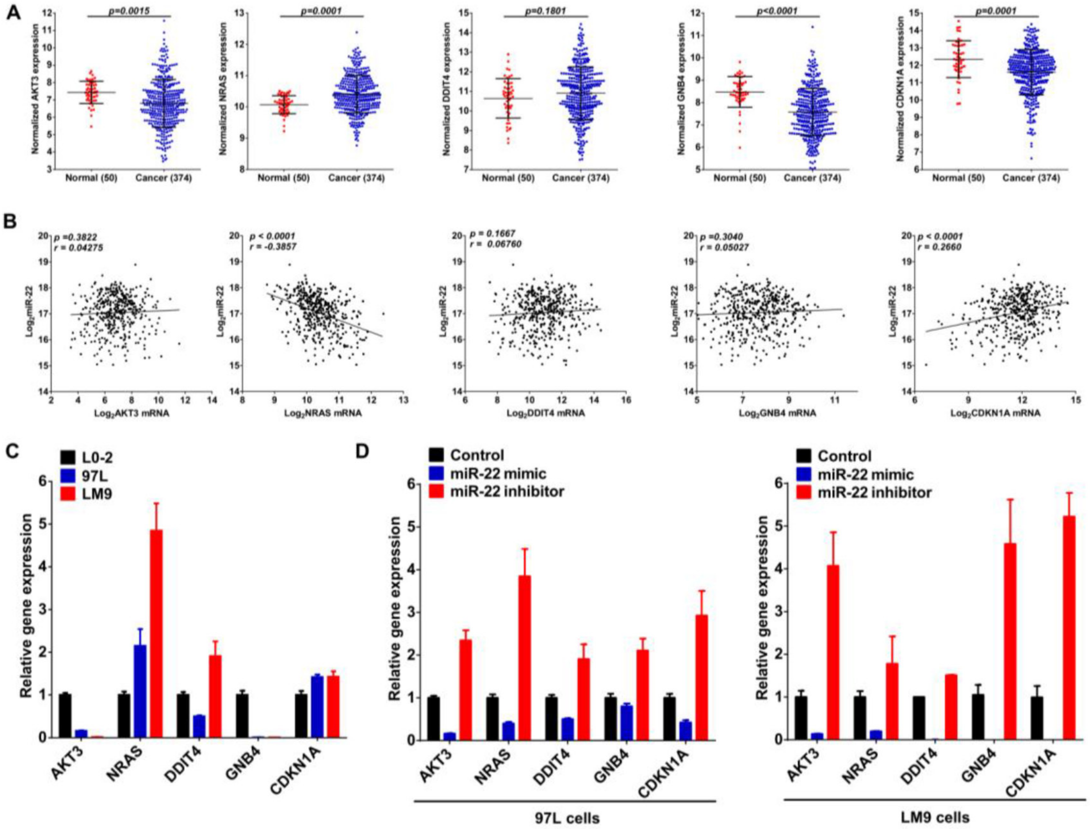

Supplementary Figure S4: miR-22 regulates mRNAs expression in HCC cells. (**A**) mRNA expression of AKT3, CDKN1A, DDIT4, GNB4 and NRAS in HCC patients. (**B**) Correlation between miR-22 and mRNA expression of these 6 transcripts. (**C**) mRNA expression of these 6 transcripts in L0-2, MHCC97L and HCCLM9 cells. (**D**) The effect of miR-22 on AKT3, CDKN1A, DDIT4, GNB4, NRAS and YWHAZ mRNA levels in MHCC97L (left panel) and HCCLM9 (right panel) cells was determined by qRT-PCR 48 h following transfection of the indicated cell line with negative control miRNA (control), miR-22 mimic or miR-22 inhibitor. Data are displayed as the Mean ± SD of three independent experiments.

Supplementary Table S1: Expression abundance of miRNAs in paired hepatocellular carcinoma tissues

miRNA	Transcript per million (TPM) miRNA mapped (Mean ± SD)				Mean fold change (tumor *vs.* normal)
	Tumor tissues (49)	Rank	Normal tissues (49)	Rank	
hsa-mir-21	202207 ± 114192	(1)	82706 ± 37638	(3)	2.4449
hsa-mir-22	144300 ± 76050	(2)	203000 ± 45550	(1)	0.7107
hsa-mir-148a	110675 ± 67282	(3)	149113 ± 35268	(2)	0.7422
hsa-mir-143	79766 ± 65259	(4)	60230 ± 27823	(4)	1.3244
hsa-mir-192	39722 ± 22367	(5)	51650 ± 9228	(5)	0.7691
hsa-mir-10b	28913 ± 29315	(6)	2083 ± 2333	(38)	13.8785
hsa-mir-122	27119 ± 19460	(7)	39548 ± 9369	(7)	0.6857
hsa-mir-30a	23775 ± 29189	(8)	31775 ± 4682	(8)	0.7482
hsa-mir-101-1	21943 ± 12971	(9)	47584 ± 11902	(6)	0.4612
hsa-mir-103-1	21872 ± 9549	(10)	16166 ± 4951	(13)	1.3530
hsa-mir-30e	19253 ± 8643	(11)	29958 ± 6689	(9)	0.6427
hsa-mir-10a	14869 ± 11767	(15)	29744 ± 10464	(10)	0.4999

Supplementary Table S2: Clinicopathological characteristics of HCC patients according to miRNAs expression

	**miR-22 expression (Mean ± SD)**	***p* value**	**miR-9-1 expression (Mean ± SD)**	***p* value**	**miR-9-2 expression (Mean ± SD)**	***p* value**
Age (years)		0.0080*		0.3620		0.3701
≥ 60	17.06 ± 0.6626		7.158 ± 2.222		7.156 ± 2.224	
< 60	16.86 ± 0.7272		6.947 ± 2.222		6.948 ± 2.219	
Gender		0.4518		0.7340		0.7210
Female	16.93 ± 0.7025		7.007 ± 2.069		7.001 ± 2.081	
Male	16.99 ± 0.6985		7.088 ± 2.296		7.090 ± 2.290	
Edmondson–Steiner grade		0.0005*		0.0554		0.0507
G1–2	17.07 ± 0.7037		6.890 ± 2.132		6.886 ± 2.137	
G3–4	16.81 ± 0.6566		7.349 ± 2.351		7.354 ± 2.340	
TNM stage		0.0003*		0.4892		0.4644
I–II	17.04 ± 0.6588		7.023 ± 2.178		7.019 ± 2.185	
III–IV	16.73 ± 0.7899		7.212 ± 2.391		7.220 ± 2.372	
AFP level (ng/ml)		< 0.0001*		0.6148		0.5929
≥ 400	16.64 ± 0.6784		7.125 ± 2.202		7.127 ± 2.203	
< 400	17.15 ± 0.5975		6.975 ±2.076		6.967 ± 2.082	
Alcohol consumption		0.5456		0.9437		0.9318
Yes	17.03 ± 0.6900		7.072 ± 2.145		7.071 ± 2.153	
No	16.98 ± 0.6869		7.055 ± 2.254		7.050 ± 2.254	
HBV/HCV infection		0.8287		0.0909		0.0926
Yes	16.98 ± 0.6468		6.866 ± 2.097		6.866 ± 2.093	
No	16.99 ± 0.7234		7.270± 2.326		7.268 ± 2.330	
Vascular invasion		0.0448*		0.0061*		0.0065*
None	17.05 ± 0.6619		6.710 ± 1.979		6.709 ± 1.981	
Micro/macro	16.89 ± 0.7315		7.409 ± 2.441		7.404 ± 2.445	

*p value less than 0.05 with significant difference between two groups.

Supplementary Table S3: The potential miR-22 target genes predicted by KEGG pathway analysis

Term Id	Term Name	Hit Genes	*P*-Value	FDR
hsa05220	Chronic myeloid leukemia	AKT3,CDKN1A,MECOM,NRAS	0.000126007	0.005292
hsa04012	ErbB signaling pathway	AKT3,CDKN1A,ERBB3,NRAS	0.000248391	0.005216
hsa04151	PI3K-Akt signaling pathway	AKT3,CDKN1A,DDIT4,GNB4,NRAS,YWHAZ	0.00122297	0.017122
hsa05214	Glioma	AKT3,CDKN1A,NRAS	0.00141609	0.014869
hsa05034	Alcoholism	GNB4,H3F3B,H3F3C,NRAS	0.00152842	0.012839
hsa05161	Hepatitis B	AKT3,CDKN1A,NRAS,YWHAZ	0.00178071	0.012465
hsa05218	Melanoma	AKT3,CDKN1A,NRAS	0.00199787	0.011987
hsa04010	MAPK signaling pathway	AKT3,MAX,MECOM,NRAS,SRF	0.00208565	0.01095
hsa05202	Transcriptional misregulation in cancer	CDKN1A,H3F3B,H3F3C,MAX	0.00276869	0.012921
hsa05215	Prostate cancer	AKT3,CDKN1A,NRAS	0.00379561	0.015942
hsa05203	Viral carcinogenesis	CDKN1A,NRAS,SRF,YWHAZ	0.00440141	0.016805
hsa05200	Pathways in cancer	AKT3,CDKN1A,MAX,MECOM,NRAS	0.00581724	0.02036
hsa04725	Cholinergic synapse	AKT3,GNB4,NRAS	0.00719472	0.023245
hsa05205	Proteoglycans in cancer	AKT3,CDKN1A,ERBB3,NRAS	0.00784687	0.023541
hsa05219	Bladder cancer	CDKN1A,NRAS	0.00788671	0.022083
hsa04110	Cell cycle	CDKN1A,STAG2,YWHAZ	0.00950284	0.024945
hsa05160	Hepatitis C	AKT3,CDKN1A,NRAS	0.0110268	0.027243
hsa05166	HTLV-I infection	AKT3,CDKN1A,NRAS,SRF	0.0139179	0.032475
hsa05213	Endometrial cancer	AKT3,NRAS	0.0144148	0.031864
hsa05223	Non-small cell lung cancer	AKT3,NRAS	0.0154866	0.032522
hsa05221	Acute myeloid leukemia	AKT3,NRAS	0.0171565	0.034313
hsa04150	mTOR signaling pathway	AKT3,DDIT4	0.0188994	0.036081
hsa05211	Renal cell carcinoma	AKT3,NRAS	0.0225979	0.041266
hsa04370	VEGF signaling pathway	AKT3,NRAS	0.0225979	0.041266
hsa04664	Fc epsilon RI signaling pathway	AKT3,NRAS	0.0245502	0.041244
hsa03018	RNA degradation	BTG1,EDC3	0.0258889	0.041821
hsa04062	Chemokine signaling pathway	AKT3,GNB4,NRAS	0.0277527	0.043171
hsa04662	B cell receptor signaling pathway	AKT3,NRAS	0.0293619	0.044043
hsa05169	Epstein-Barr virus infection	AKT3,CDKN1A,YWHAZ	0.0333773	0.04834

Supplementary Table S4: Sequences of RT-PCR primers and luciferase assay

Gene	Forward primer/sequence (5′-3′)	Reverse primer/sequence (5′-3′)
AKT3	GTGTACCGTGATCTCAAGTTGG	TTCATGGTGGCTGCATCTGT
CDKN1A	CAGACCAGCATGACAGATTTC	ATGTAGAGCGGGCCTTTGAG
DDIT4	GAGGAAGACACGGCTTACCTGG	TAGTTCTTTGCCCACCTGGC
GNB4	CTGAGCTCCCTCCAGCTGTT	GCCCCTCAGTGTACGTCTTG
NRAS	TGGAGCTTGAGGTTCTTGCT	TCGCCTGTCCTCATGTATTGG
YWHAZ	GTCGATCAGTCACAACAAGCA	CCGATGTCCACAATGTCAAGT
GAPDH	ACCACAGTCCATGCCATCAC	TCCACCACCCTGTTGCTGTA
YWHAZ 3′ UTR (Wild-type)	AAATAGCGGCCGCTTGAAATTTAT TCCTTA**<u>TTC</u>**CCTCTT**<u>GGCAGCT</u>** AATGGGCTCTTAT	TTTATCGCCGGCGAACTTTAAATAA GGAATAAGGGAGAACCGTCGATTAC CCGAGAATAGATC
YWHAZ 3′ UTR (Mutant)	AAATAGCGGCCGCTTGAAATTTATT CCTTA**<u>TTC</u>**CCTCTT**<u>AGATCAG</u>** AATGGGCTCTTAT	TTTATCGCCGGCGAACTTTAAAT AAGGAATAAGGGAGAATCTAGT CTTACCCGAGAATAGATC

Nucleotides in bold and underlined represent the putative miR-22 binding sequence.
